# A Novel Sample Selection Approach to Aid the Identification of Factors That Correlate With the Control of HIV-1 Infection

**DOI:** 10.3389/fimmu.2021.634832

**Published:** 2021-03-11

**Authors:** Julia Makinde, Eunice W. Nduati, Anna Freni-Sterrantino, Claire Streatfield, Catherine Kibirige, Jama Dalel, S. Lucas Black, Peter Hayes, Gladys Macharia, Jonathan Hare, Edward McGowan, Brian Abel, Deborah King, Sarah Joseph, The IAVI Protocol C Investigators, Eric Hunter, Eduard J. Sanders, Matt Price, Jill Gilmour

**Affiliations:** ^1^IAVI Human Immunology Laboratory, Imperial College London, London, United Kingdom; ^2^Kenya Medical Research Institute-Wellcome Trust Research Programme, Kilifi, Kenya; ^3^Department of Epidemiology and Biostatistics, MRC-PHE Centre for Environment and Health, Imperial College London, London, United Kingdom; ^4^Emory Vaccine Centre, Yerkes National Primate Research Centre, Emory University, Atlanta, GA, United States; ^5^Zambia-Emory HIV Research Project, Lusaka, Zambia; ^6^IAVI, New York, NY, United States; ^7^Department of Epidemiology and Biostatistics, University of California, San Francisco, San Francisco, CA, United States

**Keywords:** HIV-1, elite controllers, infection–immunology, viral control, immunology & infectious diseases

## Abstract

Individuals infected with HIV display varying rates of viral control and disease progression, with a small percentage of individuals being able to spontaneously control infection in the absence of treatment. In attempting to define the correlates associated with natural protection against HIV, extreme heterogeneity in the datasets generated from systems methodologies can be further complicated by the inherent variability encountered at the population, individual, cellular and molecular levels. Furthermore, such studies have been limited by the paucity of well-characterised samples and linked epidemiological data, including duration of infection and clinical outcomes. To address this, we selected 10 volunteers who rapidly and persistently controlled HIV, and 10 volunteers each, from two control groups who failed to control (based on set point viral loads) from an acute and early HIV prospective cohort from East and Southern Africa. A propensity score matching approach was applied to control for the influence of five factors (age, risk group, virus subtype, gender, and country) known to influence disease progression on causal observations. Fifty-two plasma proteins were assessed at two timepoints in the 1st year of infection. We independently confirmed factors known to influence disease progression such as the B^*^57 HLA Class I allele, and infecting virus Subtype. We demonstrated associations between circulating levels of MIP-1α and IL-17C, and the ability to control infection. IL-17C has not been described previously within the context of HIV control, making it an interesting target for future studies to understand HIV infection and transmission. An in-depth systems analysis is now underway to fully characterise host, viral and immunological factors contributing to control.

## Introduction

Individuals infected with HIV display varying rates of viral control and disease progression, with a small percentage being able to spontaneously control *in vivo* viral replication without the need for anti-retroviral treatment (ART) (1). Such exquisite control is likely to happen in the very early battle between host and virus in acute and early HIV infection (2). Our understanding of host-pathogen interactions and the mechanisms underpinning the immune response to HIV infection have been informed by studies of individuals who demonstrate an enhanced ability to control *in vivo* viral replication, and on non-pathogenic SIV infection in non-human primates (NHP) (3). However, many of the studies of HIV control are cross-sectional after set point viral load and control has been achieved. Many of these studies have been focussed in Clade A and C infection. A full understanding of the mechanisms governing such spontaneous control of infection has been hampered by the paucity of informative and linked samples coupled to technology with sufficient resolution to define this phenomenon.

Systems-based approaches have helped define novel factors driving disease progression and protection during infections such as tuberculosis (4, 5), yellow fever (6), malaria (7), and influenza (8). But their application to aid the definition of the drivers of spontaneous control in HIV has been limited. The gene signature analysis of early gut mucosal T cell responses to HIV-1 suggest that the absence of an inflammatory gene signature may define Long-term non-progressors (LTNPs) (9). But recent scRNA-Seq profiling during acute HIV infection in a limited number of treatment-naïve subjects from the Females Rising through Education, Support and Health (FRESH) (10) cohort described an interferon response gene signature before peak viraemia as well as the presence of gene modules associated with antiviral control (APOBEC3A, IFITM1, and IFITM3) in individuals able to naturally control infection (11). The *post hoc* integrated systems analysis to the RV144 trial samples also uncovered roles for Type I and II interfons, as well as IRF7 and mTORC1 in susceptibility to infection post-vaccination (12). The mammalian target of rapamycin metabolic pathway has also been shown to be key to enhanced CD8 activity in elite controllers (13). These studies highlight the potential to utilise systems methods to define the correlates associated with the control of HIV-1 infection.

Heterogeneity in the data generated using high throughput systems methodologies can be further complicated by the inherent variability encountered at the population, individual, cellular and molecular levels (14). Studies by Chowdhury et al. (13) and others (15, 16) have highlighted the diversity of transcriptional profiles that exist within a single subset of T lymphocytes that accounts in part for control of HIV infection. The control of HIV replication *in vivo* is multifactorial. Indeed viral control has been shown to be associated with age at infection, time post-infection, gender, HLA type, virus subtype and route of infection (17–22). Obtaining sufficient numbers of samples to allow for the control of all these confounders and the discovery of new correlates of disease trajectory poses a real challenge (23, 24).

**Figure 1:**
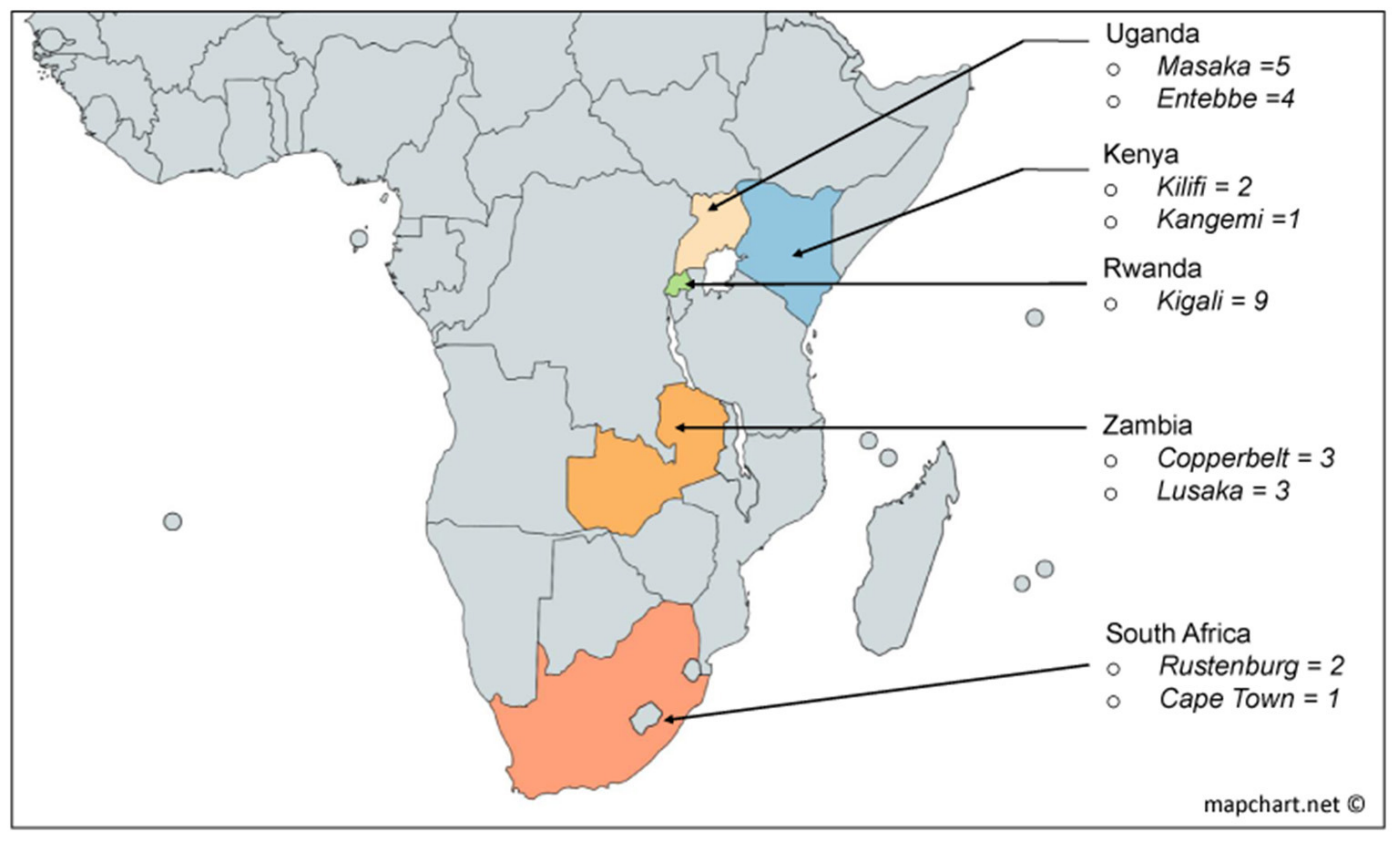
Derivation by country of the 30 volunteers used in this study from the African incidence cohort [Protocol C (22)].

We applied a unique approach to retrospectively classify HIV-infected individuals in order to aid the delineation of a profile associated with early and persistent *in vivo* control of HIV-1 replication in the absence of antiretroviral treatment. Using this approach, we defined three groups of HIV infected volunteers from Protocol C; a multisite early infection prospective cohort consisting of 613 participants recruited from nine clinical research centres in five African countries (25, 26) (Figure 1, also on https://dataspace.iavi.org/). These groups comprised volunteers with low (*n* = 10), medium (*n* = 10) and high (*n* = 10) set point viral load who were identified within days of their estimated date of HIV infection and followed over time for up to 7 years. Importantly, the low viral load volunteers showed rapid and persistent control of viral replication in the absence of treatment, and had sufficient samples available during the resolution of peak infection to enable the investigation of signatures associated with rapid and persistent HIV control. We present the profile for fifty-two soluble proteins in the acute phase of HIV infection across the three groups, demonstrating the potential to identify unique signatures associated with ART-naïve viral control using this selection approach.

## Methods

### Ethics

This study was reviewed and approved by the following ethical review boards: the Kenya Medical Research Institute Ethical Review Committee, the Kenyatta National Hospital Ethical Review Committee of the University of Nairobi, the Rwanda National Ethics Committee, the Uganda Virus Research Institute Science and Ethics Committee (Currently the UVRI Research Ethics Committee) and the Uganda National Council of Science and Technology, the University of Cape Town Health Science Research and Ethics Committee, the Bio-Medical Research Ethics Committee at the University of KwaZulu Natal, the University of Zambia Research Ethics Committee, and the Emory University Institutional Review Board. Informed consent was obtained from all volunteers prior to the collection of study related resource. All methods were carried out in accordance with relevant guidelines and regulations.

### Study Population and Selection Approach

Volunteers included in this study were selected from a historic acute and early HIV infection prospective cohort drawn from nine clinical research centres in South Africa, Zambia, Uganda, Kenya and Rwanda enrolled from 2006 to 2011 (Figure 1). Details of study characteristics, distributions, recruitment procedures, initial immunological methods and epidemiological profiling data of the Protocol C cohort are described elsewhere (18, 22, 25, 26).

Individuals from the study were ranked according to the magnitude of their mean viral load (Geometric mean) measurements taken between 9–36 months post-EDI (estimated day of infection), and divided into quartiles. Mean viral load was calculated for 362 of the 613 volunteers from the Protocol C cohort who did not receive antiretroviral treatment. A matching algorithm (27) based on the nearest neighbour was then applied to define the groups of volunteers for this study. Briefly, we selected 10 Low viral load volunteers (LVLVs) from the first quartile of the ranked dataset who had a visible period of dynamic control of infection demonstrated by the presence of a downslope in their viral load measurements, and who were able to control viral load to ≤2,000 copies/mL in the first 3 years of infection. The LVLVs were then matched with Intermediate viral load volunteers (IVLVs, *n* = 10) from the second and third quartiles, and High viral load volunteers (HVLVs, *n* = 10) from the fourth quartile of the ranked dataset. Volunteers were matched on age, clade, country, gender and risk group. Soluble proteins in plasma were assessed at two timepoints in the period following peak viraemia and within the 1st year of infection for each of the selected volunteers (Supplementary Table 1). All individuals assessed for this study were treatment naïve.

### HLA Frequency Calculation

To determine the HLA I frequencies within the 362 ART-naïve Protocol C volunteers, two-digit allelic frequencies were calculated using the Los Alamos National Laboratory HLA frequency and Graphing tool (https://www.hiv.lanl.gov/content/immunology/hla). For each MHC Class I alleles with an allele frequency >5%, we compared the set point viral load of all positive volunteers with those of all negative volunteers. Statistical tests used are described in subsequent sections.

### Plasma Analyte Quantification

Fifty-two soluble analytes were quantified in plasma using a combination of six Meso Scale Discovery (MSD) human V-PLEX panels including the Angiogenesis Panel 1 (VEGF-A, VEGF-C, VEGF-D, Tie-2, Flt-1, PIGF, bFGF), TH17 Panel 1 (IL-17A, IL-21, IL-31, IL-27, IL-23, IL-22, MIP-3α), Chemokine Panel 1 (Eotaxin, MIP-1β, Eotaxin-3, TARC, IP-10, MIP-1α, IL-8, MCP-1, MDC, MCP-4), Cytokine Panel 1 (GM-CSF, IL-1α, IL-5, IL-7, IL-12/IL-23p40, IL-15, IL-16, IL-17A, TNF-β, VEGF-A), Cytokine Panel 2 (IL-1RA, IL-3, IL-9, IL-17A/F, IL-17B, IL-17C, IL-17D, TSLP), Proinflammatory Panel 1 (IFN-γ, IL-1β, IL-2, IL-4, IL-6, IL-8, IL-10, IL-12p70, IL-13, TNF-α), and Vascular Injury Panel 2 (SAA, CRP, VCAM-1, ICAM-1). Cryopreserved plasma from the incidence study were thawed at room temperature and applied to the panels according to the manufacturer's protocol. Plates were read on the MSD plate reader model MESO QuickPlex SQ 120. All plasma samples for the study were thawed and run at the same time and grouped on plates in the order in which they were selected for the study to avoid intra-assay variability. Data was collected for two replicates per sample using the MSD software (Discovery Workbench Version 4.0). A five-parameter logistic regression formula was used to derive sample concentrations from the standard curves. Analytes below the lower limit of detection were assigned a concentration of half the lower limit of quantification (LLOQ).

### Statistical Analysis

We analysed the MSD data using a non-parametric approach, because of the small sample size and the non-Gaussian distribution, as determined using the Shapiro-Wilk test.

Non-parametric analysis (Mann-Whitney test, comparing ranks) of the differences in VL measurements for individuals expressing HLA alleles was performed in Graphpad Prism 8 Software. *P*-values <0.05 were considered significant. Propensity score matching to define study populations was executed using the MatchIT package (27) in R. Non-parametric test for similarities in the age distribution between the study groups was performed in SPSS 24.

We computed descriptive summary statistics, including the median and inter–quartile range (IQR) and Spearman correlations and excluded MIP-3α from further analyses due to missing data (40%). The null hypothesis of the difference between the two time points was assessed using Wilcoxon Signed Ranks tests. We computed the (rank based) correlation matrix for each group (LVLVs, IVLVs, HVLVs) by averaging the concentrations over time and presented the correlation matrices as heat maps.

To investigate the association between the mean viral load of volunteers (which was used to define the study groups) and the concentration of proteins in peripheral blood we fitted a linear robust regression model where a function of the ranks of the residuals was used instead of the Euclidian distance in the least square estimation (28). SAA and CRP were excluded from the model due to singularity issues. Based on these univariate results, we selected the significant proteins and estimated a multivariate robust regression model after adjusting for multiple comparison (Bonferroni). We then fitted a multivariate regression model with the significant (*p*-value adjusted 0.05) proteins and removed those that eventually where highly correlated to avoid multicollinearity issues in the regression fitting.

R analysis was conducted using version 3.1.2, 2014, (available at https://www.R-project.org).

## Results

### The Outcome of Infection Is Linked to Gender, Viral Subtype, and the Expression of Immune Receptors on Lymphocytes

**Figure 2:**
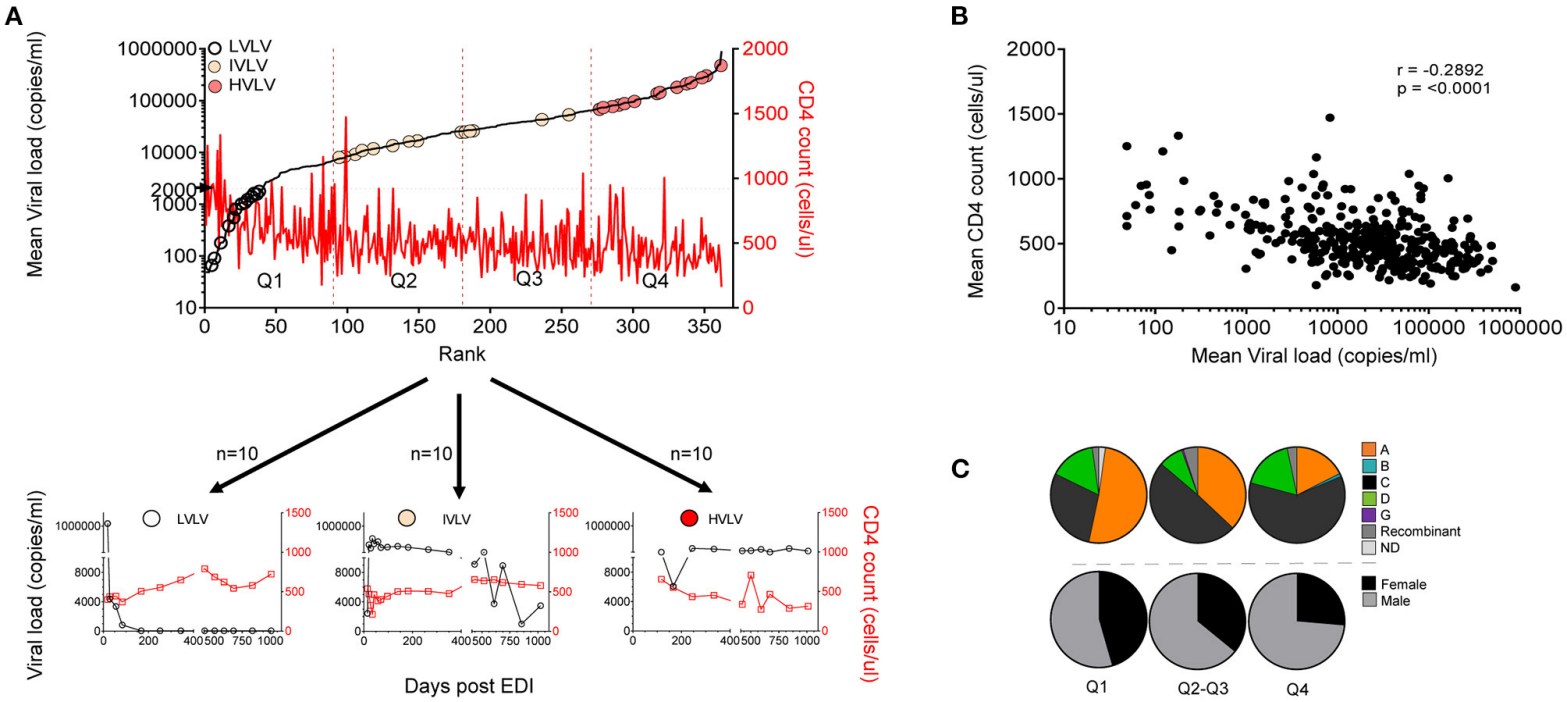
**(A)** Ranks of set point VL calculated for 362 volunteers between 9 and 36 months post infection showing distribution of Low viral load volunteers (LVLVs), Intermediate viral load volunteers (IVLVs) and High viral load volunteers (HVLVs). Ranked dataset was also divided into equal quartiles based on set point viral load. **(B)** Set point VL correlates inversely with mean CD4 counts calculated over the same period (Pearson's *r* = −0.2892; *p* < 0.0001). **(C)** Subtype and gender distribution of ART naïve volunteers by quartiles of the ranked dataset. Infecting subtype and gender both have a relationship with set point VL.

Set-point viral load represents a dynamic state of equilibrium between infecting virus and the immune response in the absence of complete elimination of the virus (29). It remains an important measure of disease progression. Set-point viral load was calculated for 362 of the 613 volunteers who did not receive antiretroviral treatment over at least 36 months of follow-up. Volunteers were then ranked and divided into equal quartiles to explore any associations with disease progression (Figure 2A). Median set point VL for the 362 volunteers was 26,061 copies/ml (IQR: 6,981–65,813 copies/mL). Mean CD4 counts calculated for the same period for all 362 volunteers was inversely correlated with mean viral load (*r* = −0.2892, *p* < 0.0001) (Figure 2B).

We examined the distribution of gender and viral subtype within our ranked dataset. In agreement with previous studies (18, 22) our analysis showed that that women had lower set point viral loads than men. There was also a higher representation of Subtype A in the lower quartiles, with the opposite being true for Subtype C infected subjects (Figure 2C).

**Figure 3:**
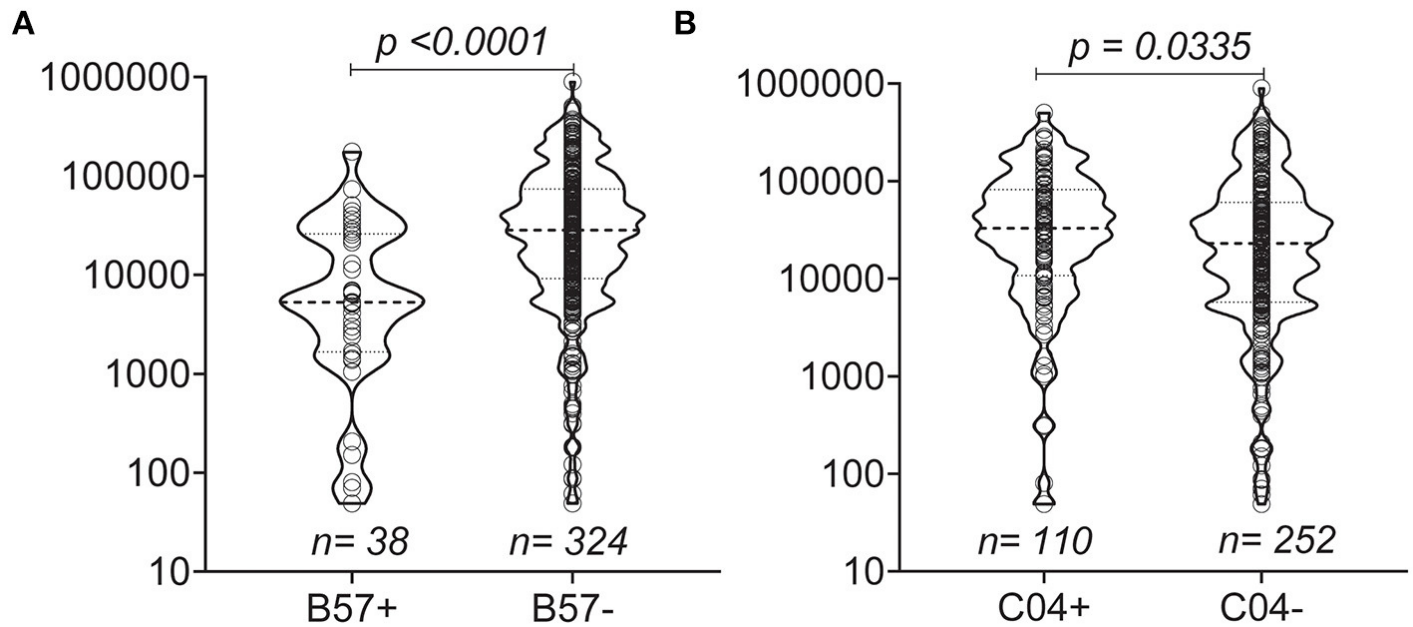
Distribution of set point VL based on the presence of MHC Class I **(A,B)**. Violin plots show individual data points as well as the 25th, 50th, and 75th percentiles. The null hypothesis was tested using a non-parametric unpaired test (Mann-Whitney *p* < 0.05 considered significant).

To assess the impact of MHC on disease progression, we compared the influence of Class I alleles with an allele frequency > 5% on the set point viral load and found that individuals with B^*^57 (*p* < 0.0001) and C^*^04 (*p* = 0.0335) had lower and higher set point viral loads, respectively, compared with individuals lacking either HLA allele (Figures 3A,B).

### A Propensity-Based Approach to Sampling an HIV Incidence Cohort to Aid Systems Analysis

**Figure 4:**
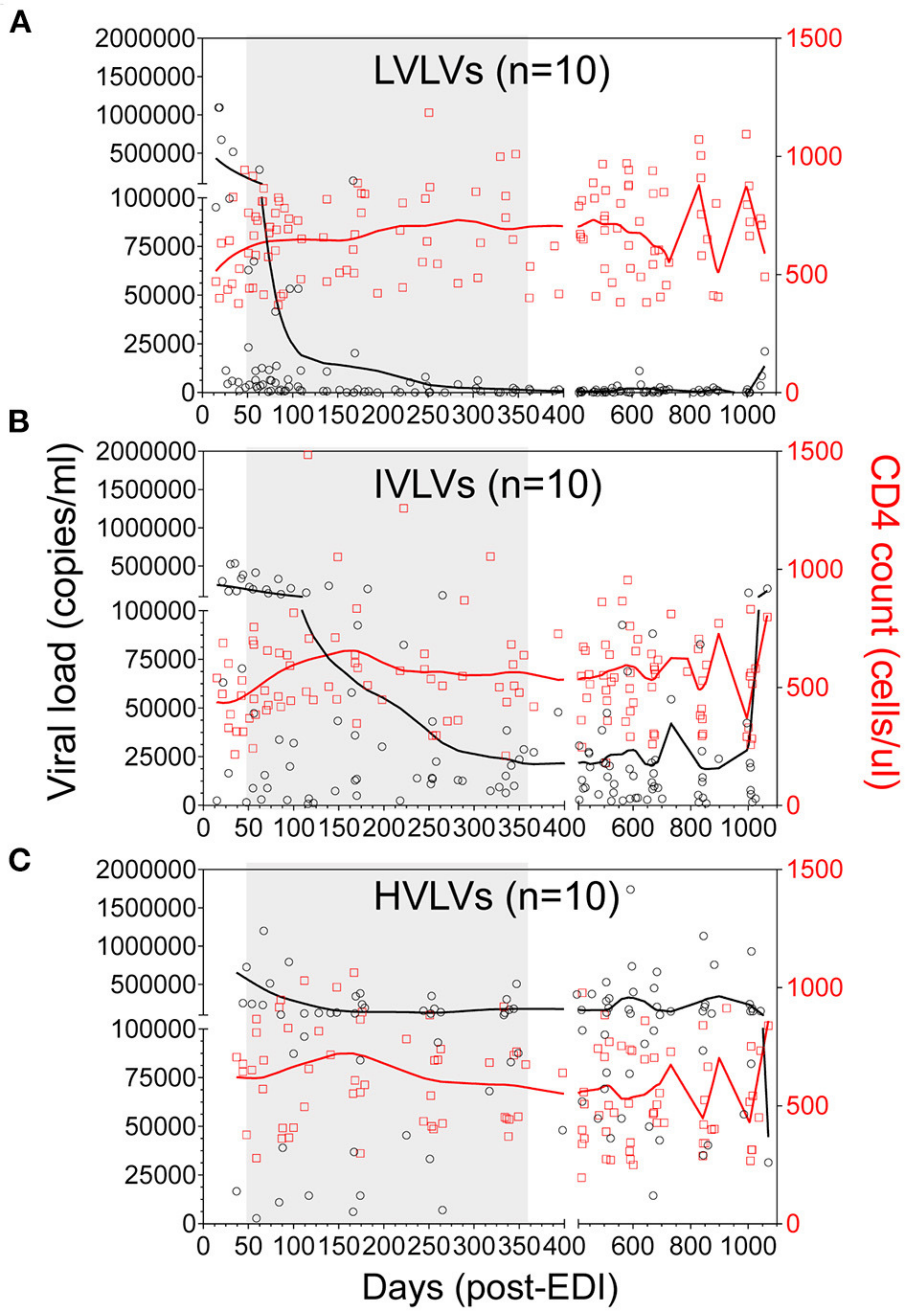
Locally weighted scatterplot smoothing (LOWESS) curves showing the overall trend in VL (black circles) and CD4 counts (red squares) for Low viral load volunteers (LVLVs) **(A)**, Intermediate viral load volunteers (IVLVs) **(B)** and High viral load volunteers (HVLVs) **(C)** over 36 months post infection. *N* = 10 for each group. The shaded region indicates the dynamic period of immune control observed in the LVLVs following peak viraemia.

During untreated HIV infection, the rate of viral replication and set-point probably reflects the dynamic interaction between the virus and host responses (23). We defined Low Viral Load Volunteers (LVLVs) as those with a set point of <2,000 copies/mL in line with previous studies (1) and were able to identify 40 Protocol C volunteers within this category. Ten of the 40 LVLVs identified had sufficient samples and viral load measurements collected over the period following peak viraemia for analysis. There was a steady decrease in viral load in the LVLVs over the 12 months following peak viraemia in the absence of ART (Figure 4A, Supplementary Figure 1). We focussed initially on the period immediately following peak viraemia in an effort to describe correlates of the early control of viral replication.

The ability to control viral replication *in vivo* has been linked to factors such as age at infection, time post-infection, gender, HLA type, route of virus entry and HIV subtype (17–22). In an effort to control for the confounding effects of some of these factors, we utilised a propensity score matching approach (27), which allows the matching of persons in one group with persons in another group based on each case's propensity score. For all the volunteers in the first group (LVLVs), we selected an equal number of volunteers from quartiles 2 and 3 (Figure 2A) based on their propensity scores for age, time post infection, gender, route of virus entry and infecting virus subtype. We designated the group selected from quartiles 2 and 3 as Intermediate viral load volunteers (IVLVs). We applied the same matching approach to volunteers in quartile 4 to identify 10 High viral load volunteers (HVLVs), from the ranked dataset. It was impossible to match on HLA haplotypes due to the diversity of alleles represented in the cohort.

We successfully identified three distinct groups consisting of 10 individuals per group, from the ranked dataset that were matched on age, gender, risk group (route of infection), country and infecting subtype (Figures 4A–C, Table 1) and for whom samples were available at two timepoints within the initial phase of control of viral replication immediately after peak viraemia. Given the age of the cohort, sample availability within this period (obtained from dataspace.iavi.org) was a real challenge. Days post EDI was also considered during the selection of these early timepoints with matched timepoints no more than 6 months apart where possible (Supplementary Table 1).

**Table 1:** Characteristics of groups selected from the HIV incidence cohort.

		**Study group**		
		**LVLVs**	**IVLVs**	**HVLVs**
Age (years)^α^		27.5 (23–32)	28.5 (28–32)	35 (29–38)
Gender	Female	5	5	5
	Male	5	5	5
Risk group	Discordant couple	8	8	8
	MSM	1	1	1
	Other heterosexual	1	1	1
Clade	A1	5	5	5
	C	3	3	3
	D	2	2	2
Country	Kenya	1	1	1
	Rwanda	3	3	3
	South Africa	1	1	1
	Uganda	3	3	3
	Zambia	2	2	2
Mean VL		909.86 (86.16–1426.02)	12744.535 (9163.02–25803.85)	114331.03 (81899.77–268402.30)
Mean CD4 count		710.345 (561.37–817.22)	533.955 (424.95–584.08)	506.085 (365.42–692.21)

*Continuous variables shown as median (interquartile range), other variables expressed as count*.

α *Wilcoxon test (p = 0.183)*.

### Concentrations of Soluble Markers in the Acute Phase Are Associated With Early and Sustained Control of *in vivo* Viral Load

We measured the levels of 52 soluble proteins in plasma at two timepoints following peak viraemia and report the median and IQR for the two timepoints for all volunteers (Table 2). For the most part, protein concentrations were not significantly different across the two early timepoints assessed with the exception of VCAM1 (*p* = 0.02) and IL-10 (*p* = 0.012) for the overall dataset, IL-6 (*p* = 0.037) and IL-17C (0.006) for LVLVs, SAA (*p* = 0.027) and CRP (*p* = 0.027) for IVLVs, and IFN-γ (*p* = 0.01) for HVLVs (group data also shown in Supplementary Figures 2, 3).

**Table 2:** Descriptive statistics for all the proteins assessed.

	**A**				**B**	**C**	**D**	**E**
**Protein**	**Time 1 (pg/mL)**		**Time 2 (pg/mL)**		**Wilcoxon Signed Ranked Test** ***p*****-values**			
	**Median**	**IQR**	**Median**	**IQR**	**Overall**	**LVLVs**	**IVLVs**	**HVLVs**
GM-CSF	0.247	0.154	0.252	0.148	0.629	0.432	0.61	0.695
IL-1α	0.318	0.961	0.13	0.499	0.162	0.236	0.624	0.722
IL-5	0.755	1.022	0.767	0.886	0.339	0.77	0.492	0.193
IL-7	1.279	0.573	1.306	1.016	0.792	0.695	0.695	0.922
IL-12/IL-23p40	84.18	86.74	72.94	61.74	0.164	0.432	1	0.084
IL-15	1.494	1.914	1.355	2.045	0.502	0.919	0.636	0.407
IL-16	216.6	118.9	205.1	148.5	0.641	0.846	0.625	0.232
TNF	0.164	0.102	0.159	0.08	0.175	0.103	0.922	0.492
IFN-γ	5.577	5.834	6.08	3.984	0.245	0.695	0.77	**0.01**
IL-1β	0.112	0.06	0.119	0.053	0.764	0.141	1	0.636
IL-2	0.403	0.356	0.303	0.307	0.202	0.695	0.636	0.16
IL-4	0.041	0.035	0.043	0.062	0.665	0.492	0.193	0.636
IL-6	0.851	0.701	1.074	0.83	0.245	**0.037**	0.232	0.131
IL-10	0.992	1.409	0.706	0.808	**0.012**	0.064	0.492	0.084
IL-12p70	0.143	0.291	0.169	0.245	0.863	0.432	0.922	0.407
IL-13	0.69	4.281	0.974	2.534	0.903	0.557	0.846	0.625
IL-1Rα	106.6	97.5	96.12	89.15	0.262	0.492	0.922	0.131
IL-17A	3.058	2.193	2.802	1.457	0.171	0.557	0.625	0.084
IL-17AF	2.043	2.281	2.143	1.428	0.968	0.77	0.77	0.625
IL-17B	0.952	0.684	1.058	0.894	0.428	1	1	0.084
IL-17C	3.065	4.577	1.943	4.364	0.07	**0.006**	0.557	0.922
IL-17D	14.68	10.25	13.69	9.831	0.761	0.625	0.557	0.625
Eotaxin	99.99	37.99	91.61	67.6	0.584	1	0.846	0.322
MIP-1β	24.13	13.78	25.5	20.39	0.871	0.922	0.695	0.846
Eotaxin3	14.73	11.03	13.9	12.95	0.67	0.846	0.625	0.557
TARC	42.4	36.19	39.67	37.17	0.109	0.432	0.432	0.375
IP-10	264.3	265.9	258.3	211	0.984	0.625	0.131	0.193
MIP-1α	9.032	4.086	8.558	4.78	0.655	0.232	0.77	1
IL-8	138.8	102.5	108.4	106.3	0.99	1	0.722	0.922
MCP-1	80.6	29.55	76.76	35.29	0.516	0.492	0.846	0.77
MDC	666.7	263.8	711.3	434.7	0.245	0.492	0.625	0.695
MCP-4	17.2	9.422	17.08	10.46	0.371	0.131	0.77	0.625
MIP-3α	7.554	4.884	5.984	4.640	–	–	–	–
VEGF	11.01	6.808	10.61	5.739	0.67	1	0.77	0.922
VEGFC	7.471	13.21	10.14	18.31	0.221	0.695	0.625	0.322
VEGFD	154.6	204.5	127.7	196.1	0.328	0.77	0.846	0.492
Tie2	2636	3500	2644	4066	0.777	0.232	0.625	0.922
Flt-1	16.54	36.8	19.7	31.68	0.503	0.846	0.922	0.275
PIGF	0.585	2.367	0.734	2.384	0.73	0.625	0.492	0.846
βFGF	0.592	1.014	0.67	1.435	0.213	0.432	0.232	1
SAA	1219000	2977000	1384000	13780000	0.135	0.375	**0.027**	0.492
CRP	1029000	3681000	1455000	6862000	0.158	0.432	**0.027**	0.432
VCAM1	658600	334200	617900	168700	**0.02**	0.492	0.16	0.16
ICAM1	518300	287700	551800	258800	0.839	0.922	0.695	1
IL-3	6.858	7.636	6.777	6.496	0.57	0.432	0.322	0.432
IL-9	0.502	0.415	0.476	0.299	0.213	0.922	0.432	0.193
TSLP	0.75	0.488	0.706	0.413	0.158	0.064	0.432	0.695
IL-21	1.723	2.404	0.872	1.828	0.08	0.286	0.183	0.636
IL-31	0.117	0.123	0.088	0.104	0.114	0.131	0.322	1
IL-27	1425	431.2	1554	677	0.855	1	0.557	0.232
IL-23	0.359	8.859	0.359	3.284	0.74	0.813	0.371	1
IL-22	1.61	5.837	2.356	6.818	0.641	0.322	0.625	0.492

*(A): Median and IQR shown at each timepoint for all 30 volunteers. (B–E): The null hypothesis of the difference between the two time points assessed was assessed using Wilcoxon Signed Ranks tests for all volunteers (B), LVLVs (C), HVLVs (D) and HVLVs (E). p-values below 0.05 were interpreted to be significant. Proteins are presented by broad functional groups. Bold values indicate where p values are less than 0.05*.

To further investigate the association between the mean viral load of volunteers (which was used to define the study groups) and the concentration of proteins in peripheral blood we applied a univariate regression model where a function of the ranks of the residuals was used instead of the Euclidian distance in the least square estimation (28). The estimates for nine analytes that were significant are shown in Table 3 (GM-CSF, *p* < 0.001; MIP 1-α, *p* < 0.001; IL-8, *p* < 0.001; IFN-γ, *p* < 0.001; IL-2, *p* < 0.001; IL-13, *p* = 0.05; IL-17C, *p* < 0.001; IL-9 = 0.02; IL-31 = 0.02).

**Table 3:** Model estimates from the univariate non-parametric model to investigate the association between the mean viral load of volunteers and the concentration of proteins in peripheral blood.

**Protein**	**Beta- coefficient**	**Standard error**	***t*-value**	***P*-value**
GM-CSF	1	0	6.55E+14	<0.001
MIP1α	124.9	34.75	3.59	<0.001
IL-8	433	64.91	6.67	<0.001
IFN-γ	25,720	7,732	3.33	<0.001
IL-2	82,300	26,460	3.11	<0.001
IL-13	31,910	15,510	2.06	0.05
IL-17C	2,721	352.3	7.72	<0.001
IL-9	3,130	1,267	2.47	0.02
IL-31	563.1	231.7	2.43	0.02

*Only proteins with a significant association with set point viral load are reported*.

Based on the results of the univariate analyses, we generated a multivariate robust regression model using the proteins which were associated with mean viral load, after adjusting for multiple comparison (Bonferroni) and excluding highly correlated analytes to avoid issues of multicollinearity. The only protein that remained significantly associated with mean viral load was MIP1-α (*p* < 0.001) after *p*-value adjustment (Holmmel). IL-8 was excluded from the model because it was highly correlated with GM-CSF (Spearman correlation, 0.71).

**Figure 5:**
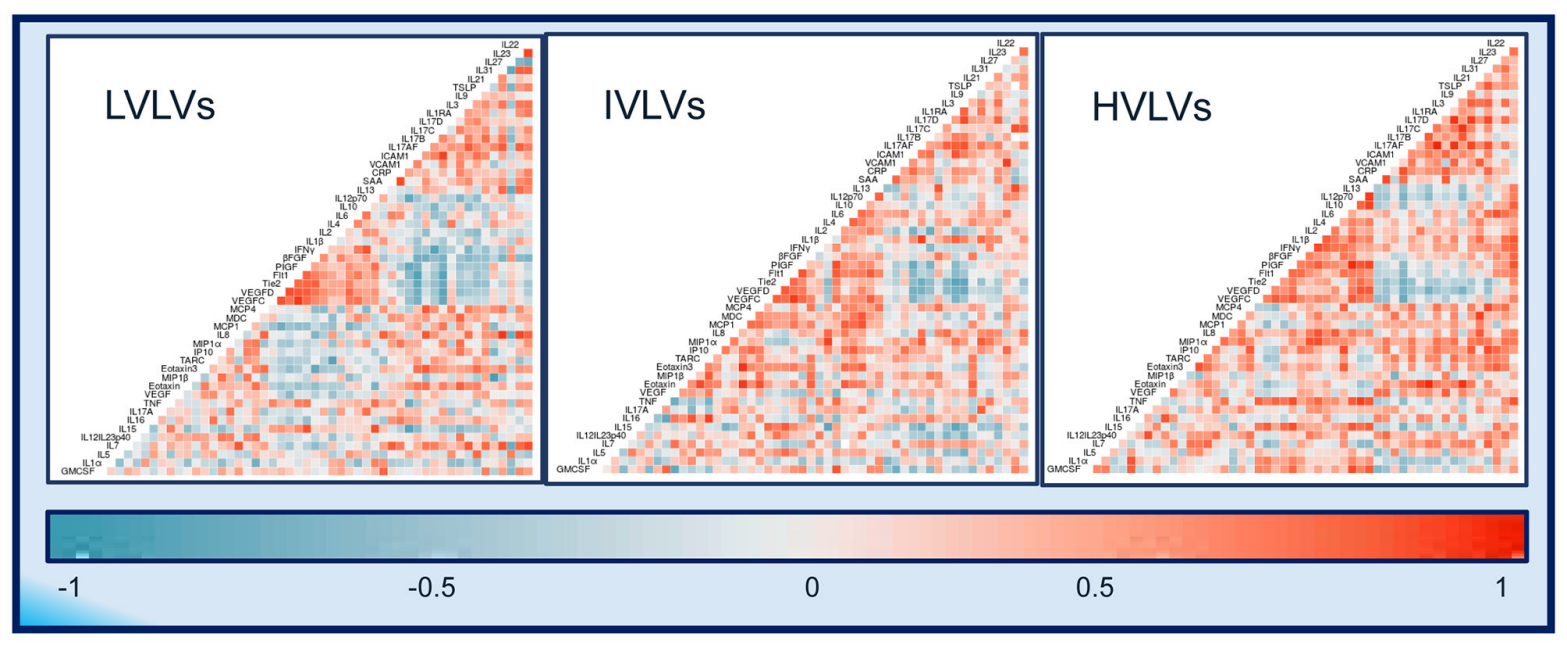
Correlograms of the correlations between 52 plasma protein concentrations for Low viral load volunteers (LVLVs), Intermediate viral load volunteers (IVLVs) and High viral load volunteers (HVLVs). Blue and red squares represent positive and negative correlations, respectively with darker colours indicating a greater magnitude of correlation.

Exploratory heatmaps based on the lower triangle Spearman correlation matrices and using the average plasma protein value between the two timepoints for each group suggests that differences exist in the relationships between different plasma proteins across the groups (Figure 5) with more frequent positively correlated proteins seen in IVLVs and HVLVs compared to LVLVs.

## Discussion

We present a unique approach to classifying individuals drawn from an acute and early HIV infection cohort that considers a range of factors known to have an impact on disease progression, to efficiently define the peripheral secretory profile associated with early and sustained control of *in vivo* viral replication. This selection approach enabled us to define the profile of 52 proteins deployed within the specific period of dynamic immunological control of viral replication for all volunteers in the absence of antiretroviral treatment. Expectedly our ranking approach show that measurements for CD4 cells, which are the first cells to become infected during transmission (30, 31) and continue to be a primary target for HIV-1 (32), were negatively correlated with set point viral load calculations.

HIV subtype has been shown to be associated with disease trajectory and outcome (21, 22, 33, 34) but limitations of study size and design have meant that such findings have been largely descriptive in nature. By matching the selected individuals from Protocol C based on other relevant confounding variables, we were able to address the independent contribution of viral subtype to the control of viral replication *in vivo*. We report similar observations to Price et al. (22) who performed a subtype-by-geographic-region covariate analysis on the whole Protocol C cohort and showed that Subtype A-infected volunteers were more likely to control viral load than Subtype-C infected volunteers. Also in this cohort, Amornkul et al. (18) show that subtype C is associated with faster progression to AIDS and CD4+ T cell decline compared to subtype A. Here we also show a higher representation of Subtype-A relative to Subtype-C infected subjects in the lower quartiles of the viral load-ranked ranked dataset of the same cohort.

Whilst the diversity of HLA types represented in the cohort did not permit complete matching of volunteers based on this factor, well-reported trends like the favourable influence of B^*^57 on disease control were evident. The presence of the less studied C^*^04 HLA Class I allele, which is reportedly associated with B^*^35 on chromosome 6 (35, 36) appeared to have a less favourable influence on disease control in this study. This potentially deleterious effect of the C^*^04 allele on disease progression has only been reported by a few studies that focused on either HIV-1 subtype B alone (35–37) or mostly C (38). Our data covering HIV-1 subtype A, C, D and recombinant viral subtypes suggests that this effect of C^*^04 may apply regardless of the subtype of the virus. It has been suggested that this effect may be mitigated by the association of C^*^04 with other deleterious HLA Class I alleles or with the killer-cell immunoglobulin-like receptor (KIR) KIR2DS4 (37). These observations provide some validation of the novel propensity matching method presented in this study.

Early HIV infection is characterised by a cytokine storm that is detectable at the levels of gene (11) and protein expression (39). We were able to focus on the dynamics of the plasma protein response following peak viraemia and during the 1st year of infection when the immune system is most actively involved in the control of viral replication. The fact that the circulating levels of most of the plasma proteins appeared to be largely stable in all volunteers with a few exceptions (IL-6, IL-17C, SAA, CRP, and IFN-γ) over the period assessed was somewhat surprising. For the most part the indicated analytes have been linked previously to HIV disease outcomes (40, 41), even if the strength of their associations remain poorly understood. A notable exception was the falling levels of IL-17C observed in LVLVs. In contrast to other members of the IL-17 family (IL-17A and IL-17F), IL-17C is predominantly produced by epithelial cells (42–44) and not leucocytes (45), with broad activity on epithelial cells, TH17 leucocytes (46) and monocytic lineage cells (43). Although not fully described within the context of HIV-1 infection, early release of IL-17C in other models of infection suggest a dual function in the regulation of both innate and adaptive immune responses (45). The decreasing levels of serum IL-17C seen in LVLVs may suggest a role in early recruitment and differentiation of innate and adaptive modulators in response to HIV infection-i.e., prior to peak viral load, and subsequent downregulation in those who eventually go on to control infection. The difference in its cellular source compared to other members of the IL-17 cytokine family makes it a potentially interesting target for further studies to examine a potential role in barrier immunity during and immediately after transmission at mucosal surfaces.

Our regression analysis suggests a relationship between the levels of nine plasma proteins including IL-17C in the period following peak viraemia and set point viral load, with MIP-1α being the most significantly associated with mean viral load in the multivariate analysis. MIP-1α is one of three well-characterised β-chemokines produced by immune cells including CD8 and CD4 T cells that have been implicated in the inhibition of HIV infection (47, 48). Whilst the evidence strongly suggests that T cell capacity to secrete β-chemokines like MIP-1α is strongly associated with *in vivo* and *in vitro* viral control (49–52), the relationship between the quantity of circulating soluble β chemokines and disease outcome appears to be less clear (53–55). Our results suggest that the concentration of MIP-1α in the period following peak viraemia is strongly associated with set point viral load.

Several groups have assessed the relationships between specific cytokines and the control of viral replication (56–58). Our exploratory analysis of the correlations between 52 proteins during the period following peak viraemia suggest that disease progression is underpinned by clear differences in the deployment of immune modulators. Whilst the functional implications of the negatively correlated proteins in LVLVs is not immediately clear, this and previous observations validate the unique selection approach presented here and the potential to apply it to support systems investigation of the correlates that define natural control of HIV-1 infection.

The impact of biological sex on the outcome of viral infections has been highlighted by other groups (59, 60). Our ranked data set showed that women had lower set point viral loads than men. In comparing the two groups (men and women), we identified differences in the levels of IL-7, a cytokine implicated in early T cell development, proliferation and differentiation (61) in the period studied. Levels of thymus and activation-regulated chemokine (TARC), a chemokine constitutively produced in the thymus and by keratinocytes and dendritic cells (62, 63) with powerful chemoattractant effect on T cells was also found to be higher in men in this study. Whilst recent studies examining these sex differences point to a possible role for increased levels of innate inflammatory cytokines (60) on disease progression during viral infections, our data may indicate differences in how T cells are activated/recruited and deployed in men and women. A recent study by El-Badry et al. highlights the impact of plasma levels of 17β-estradiol in women on T cell activation in the acute phase of HIV infection. It is worth noting the small sample size from which these observations were made; confirmation of our observations will be required.

Taken together with previous results, it is reasonable to state that whilst our results suggest an association between levels of soluble MIP-1α in the period of active immune suppression of viral replication and disease progression, they also support the notion that the mechanism of *in vivo* suppression of HIV is likely multifactorial (64). As such efforts aimed at reducing the potential for noise in datasets will go a long way to enable the definition of the correlates associated with immunological control of HIV.

We present our unique selection approach as a way to potentially counter some of the noise associated with extreme heterogeneity in datasets allowing for the application of high-resolution systems methodologies to define the correlates associated with natural control of HIV infection. Whilst the ranking and propensity-based selection methods presented may not directly predict correlates of natural protection against HIV-1, they enable the exclusion of any noise arising as a result of the factors that are controlled for in the study design. Given that HIV pathogenesis is multifactorial, the tendency for such noise to obscure valid observations is considered a real barrier to the application of high dimensional (or systems) analytical methods to aid the definition of the correlates of natural control (14). We demonstrate the utility of the ranking and matching approach by independently confirming known factors associated with disease progression, albeit in a small study population. We also identify novel soluble proteins such as IL-17C and illustrate differences in the pattern of deployment of peripheral cytokines that tally with disease progression. These findings demonstrate the utility of the unique ranking and selection approach for systems analysis is subsequent studies.

## Data Availability Statement

The raw data supporting the conclusions of this article will be made available by the authors, without undue reservation.

## Ethics Statement

This study was reviewed and approved by the following ethical review boards: the Kenya Medical Research Institute Ethical Review Committee, the Kenyatta National Hospital Ethical Review Committee of the University of Nairobi, the Rwanda National Ethics Committee, the Uganda Virus Research Institute Science and Ethics Committee (Currently the UVRI Research Ethics Committee) and the Uganda National Council of Science and Technology, the University of Cape Town Health Science Research and Ethics Committee, the Bio-Medical Research Ethics Committee at the University of KwaZulu Natal, the University of Zambia Research Ethics Committee, and the Emory University Institutional Review Board. Informed consent was obtained from all volunteers prior to the collection of study related resource. All methods were carried out in accordance with relevant guidelines and regulations. The patients/participants provided their written informed consent to participate in this study.

## Author Contributions

JM was responsible for conceptualisation, methodology, formal analysis, data curation, sample application preparation, original draft preparation, review, and editing. EN was responsible for methodology development, sample application preparation, and original draft review. AF-S was responsible for statistical analysis. CS was responsible for running immunological assays. CK, JD, SB, GM, and PH were responsible for methodology development and original draft review. JH was responsible for assay review, processing and approval of sample application. DK, SJ, EM, and BA were responsible for methodology development, data curation, assay review and original draft review. EH, MP, ES, and JG were responsible for project conceptualisation, methodology development, data curation, granting sample access, review, and editing. The IAVI protocol C investigators were responsible for the initiation and successful completion of the Protocol C study. All authors contributed to the article and approved the submitted version.

## List of the IAVI Protocol C Investigators

- Eduard J. Sanders, Centre for Geographic Medicine -Coast/KEMRI, Kenya; University of Oxford, UK.
- Omu Anzala, Kenya AIDS Vaccine Institute -Institute of Clinical Research, Kenya.
- Anatoli Kamali, Medical Research Council/Uganda Virus Research Institute, Uganda Research Unit on AIDS, Uganda.
- Etienne Karita, Project San Francisco, Rwanda.
- William Kilembe, Mubiana Inambao, Shabir Lakhi, Zambia Emory Research Project, Zambia.
- Susan Allen, Eric Hunter, Emory University, Georgia, USA.
- Vinodh Edward, The Aurum Institute, South Africa.
- Pat Fast, IAVI, New York, USA.
- Matt A. Price, IAVI, New York, USA; Department of Epidemiology and Biostatistics, University of California San Francisco, USA.
- Jill Gilmour, IAVI Human Immunology Laboratory, Imperial College, UK.
- Jianming Tang, Ryals Public Health Building, University of Alabama, USA.
- Fran Priddy, IAVI, New York, USA.
- Mary H. Latka, The Aurum Institute, South Africa.
- Linda-Gail Bekker, Desmond Tutu HIV Foundation, South Africa.

## Conflict of Interest

The authors declare that the research was conducted in the absence of any commercial or financial relationships that could be construed as a potential conflict of interest.
