# Supplementary material for: A Novel Sample Selection Approach to Aid the Identification of Factors That Correlate With the Control of HIV-1 Infection
Source: Front Immunol. 2021 Mar 11;12:634832. doi: 10.3389/fimmu.2021.634832 (PMC7991997; doi:10.3389/fimmu.2021.634832)
Supplement: Supplementary file 1 [file Data_Sheet_1.docx]

Supplementary Material


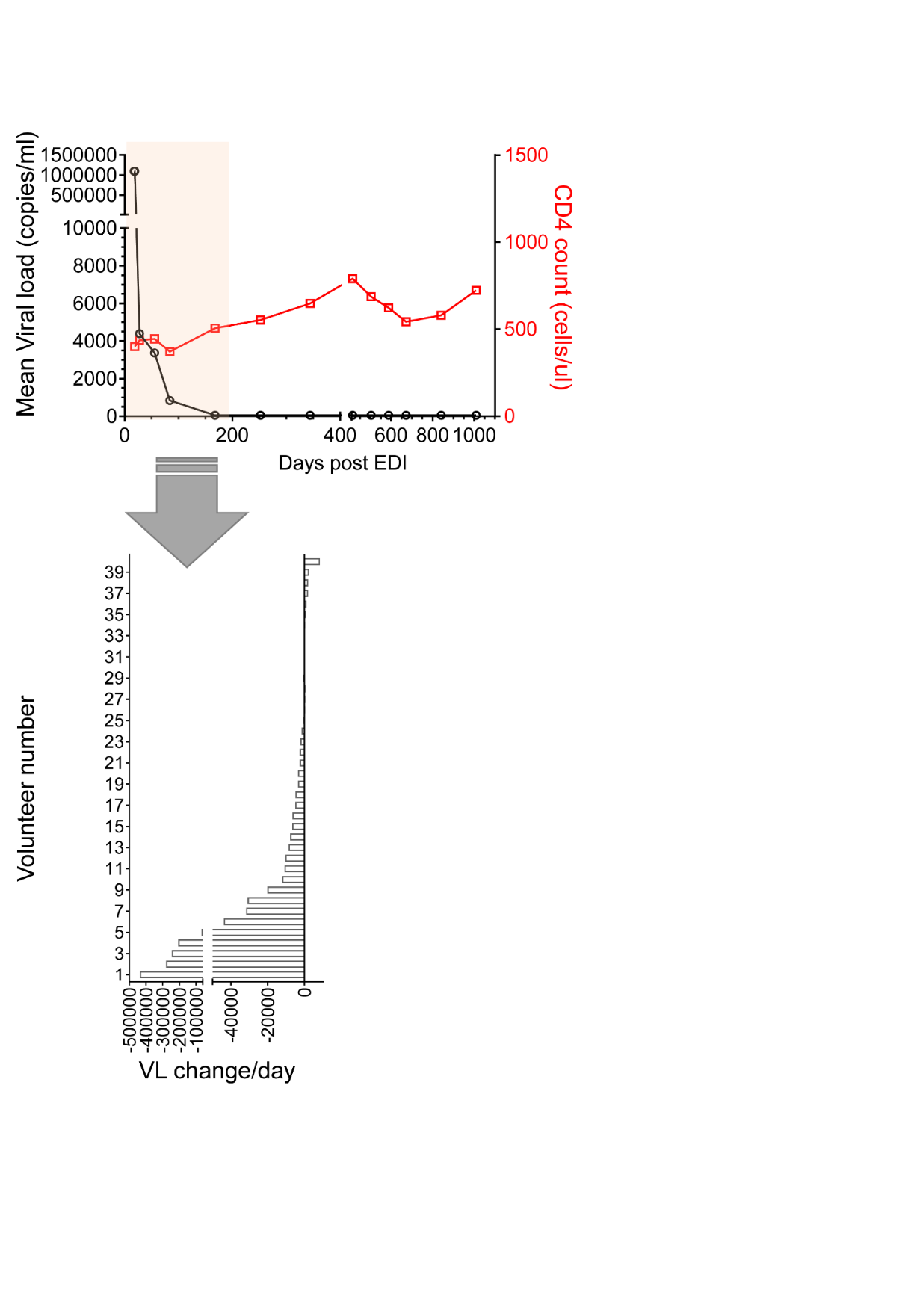


Figure S1: 40 Low viral load volunteers (LVLVs) from the cohort were identified based on set point VL calculations ≤2000 copies/ml and the presence of a negative slope after peak viraemia. Top graph shows the typical VL curve for a controller. Bottom graph shows the rate of change in VL (VL change/day) for all 40 LVLVs using viral load measurements taken over the first 18 months post infection.


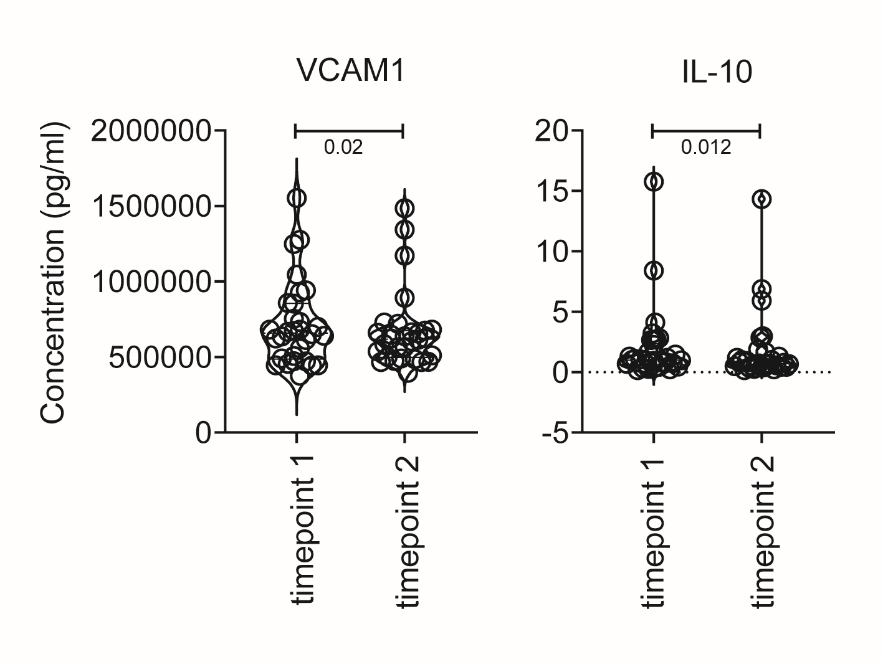


Figure S2: Cytokines found to be significantly different between the two timepoints tested for the entire dataset. The null hypothesis of difference between the two timepoints was tested using Wilcox Signed Ranks tests for matched comparisons.


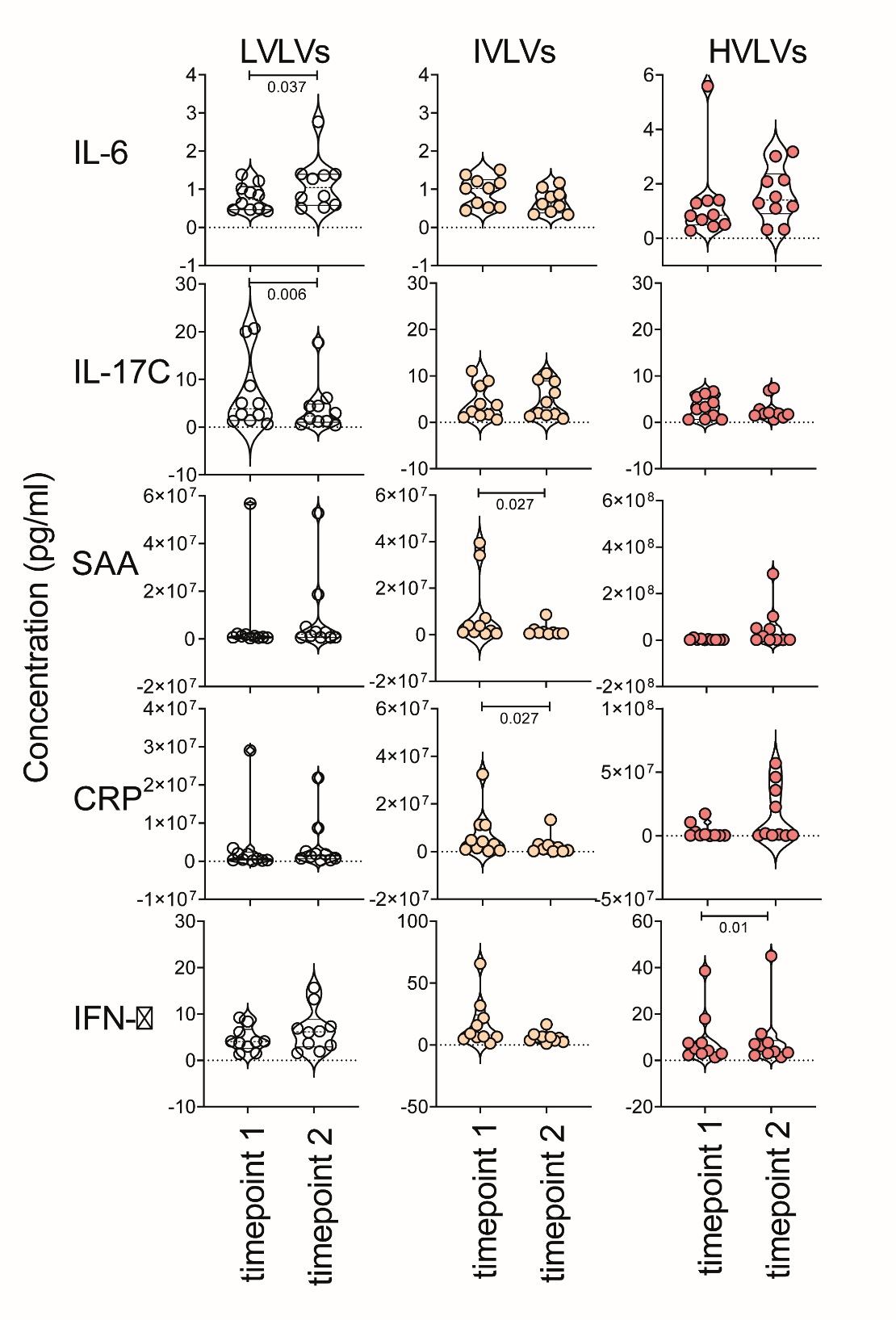


Figure S3: Cytokines found to be significantly different between the two timepoints tested for Low viral load volunteers (LVLVs), Intermediate viral load volunteers (IVLVs) and High viral load volunteers (HVLVs). The null hypothesis of difference between the two timepoints was tested using Wilcox Signed Ranks tests for matched comparisons.

Figure S4: Cytokines found to be significantly different between the men and women regardless of their study groupings. A total of 15 men and 15 women were compared. The null hypothesis of difference between the two timepoints was tested using Wilcox Signed Ranks tests for matched comparisons.


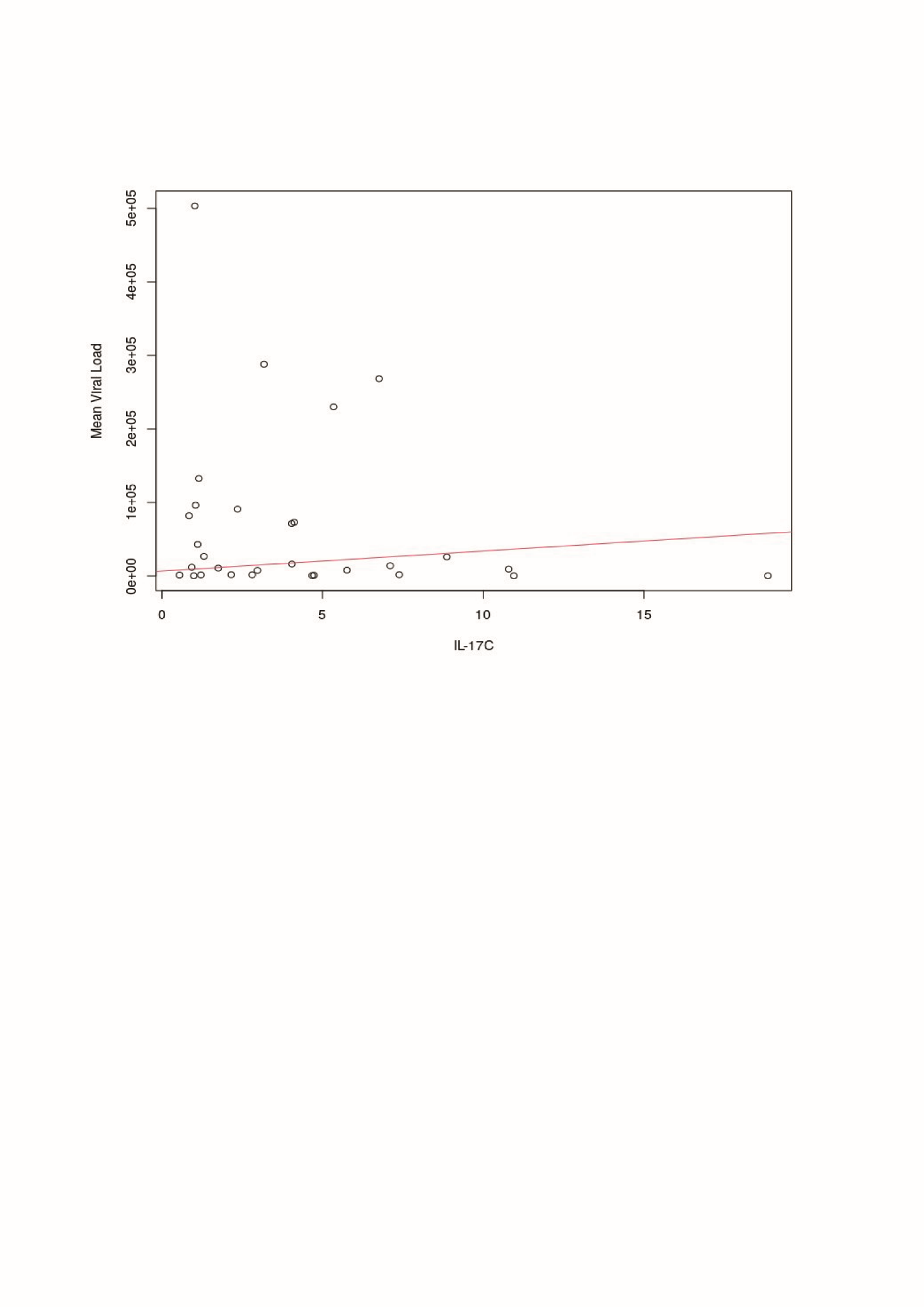


Figure S5: IL-17C regression analysis to determine association with mean viral load.

Supplementary Table 1: We defined three groups of individuals (LVLVs, IVLVs, and HVLVs) from the ranked dataset that were matched on age, gender, risk group (route of infection), country and infecting subtype (Figure 4 A-C, Table 1) and for whom samples were available at two timepoints within the dynamic phase of immunological control following peak viraemia. Days post EDI was considered in selecting the acute timepoints for analysis with matched timepoints no more than six months apart where possible

| Test set | Mean VL | Mean CD4 counts | Age | Gender | Clade | Country | Risk group | Study group | Timepoint 1  (Days post EDI) | Timepoint 2  (Days post EDI) |
| --- | --- | --- | --- | --- | --- | --- | --- | --- | --- | --- |
| 1 | 49.00 | 635.37 | 29 | Male | A | Rwanda | Discordant couple | LVLV | 84 | 168 |
|  | 16115.85 | 587.20 | 28 | Male | A | Rwanda | Discordant couple | IVLV | 42 | 265 |
|  | 72996.71 | 425.69 | 23 | Male | A | Rwanda | Discordant couple | HVLV | 166 | 244 |
| 2 | 1589.43 | 817.22 | 30 | Female | C | Zambia | Discordant couple | LVLV | 249 | 347 |
|  | 7399.93 | 249.46 | 31 | Female | C | Zambia | Discordant couple | IVLV | 170 | 254 |
|  | 96152.19 | 365.42 | 29 | Female | C | Zambia | Discordant couple | HVLV | 265 | 349 |
|  | 70.21 | 945.58 | 21 | Female | C | South Africa | Other heterosexual | LVLV | 21 | 176 |
| 3 | 10522.98 | 412.68 | 21 | Female | C | South Africa | Other heterosexual | IVLV | 71 | 336 |
|  | 90791.55 | 624.66 | 36 | Female | C | South Africa | Other heterosexual | HVLV | 112 | 256 |
|  | 758.28 | 778.60 | 23 | Male | A | Rwanda | Discordant couple | LVLV | 169 | 247 |
| 4 | 7753.82 | 584.08 | 48 | Male | A | Rwanda | Discordant couple | IVLV | 93 | 258 |
|  | 268402.30 | 692.21 | 53 | Male | A | Rwanda | Discordant couple | HVLV | 176 | 260 |
|  | 1426.02 | 420.36 | 26 | Male | A | Rwanda | Discordant couple | LVLV | 222 | 395 |
| 5 | 42799.08 | 829.85 | 27 | Male | A | Rwanda | Discordant couple | IVLV | 222 | 318 |
|  | 71206.27 | 705.91 | 41 | Female | A | Rwanda | Discordant couple | HVLV | 141 | 317 |
|  | 398.36 | 561.37 | 23 | Male | D | Kenya | MSM | LVLV | 73 | 252 |
| 6 | 13836.40 | 552.41 | 28 | Male | D | Kenya | MSM | IVLV | 146 | 287 |
|  | 230002.07 | 343.70 | 28 | Male | D | Kenya | MSM | HVLV | 84 | 253 |
|  | 1595.43 | 798.56 | 26 | Female | A | Uganda | Discordant couple | LVLV | 176 | 219 |
| 7 | 11652.67 | 515.50 | 28 | Female | A | Uganda | Discordant couple | IVLV | 198 | 282 |
|  | 288052.23 | 557.20 | 35 | Female | A | Uganda | Discordant couple | HVLV | 179 | 347 |
|  | 1278.85 | 432.65 | 44 | Male | C | Zambia | Discordant couple | LVLV | 108 | 194 |
| 8 | 9163.02 | 501.77 | 41 | Male | C | Zambia | Discordant couple | IVLV | 96 | 168 |
|  | 503486.43 | 365.30 | 38 | Male | C | Zambia | Discordant couple | HVLV | 174 | 338 |
|  | 86.16 | 874.28 | 40 | Female | D | Uganda | Discordant couple | LVLV | 252 | 336 |
| 9 | 26580.32 | 424.95 | 31 | Female | D | Uganda | Discordant couple | IVLV | 262 | 341 |
|  | 81899.77 | 874.02 | 35 | Female | D | Uganda | Discordant couple | HVLV | 167 | 333 |
|  | 1061.43 | 642.09 | 32 | Female | A | Uganda | Discordant couple | LVLV | 222 | 308 |
| 10 | 25803.85 | 555.89 | 29 | Female | A | Uganda | Discordant couple | IVLV | 168 | 245 |
|  | 132509.87 | 454.97 | 33 | Male | A | Uganda | Discordant couple | HVLV | 168 | 252 |
